# Quantitative Evaluation of Compliance with Recommendation for Sulfonylurea Dose Co-Administered with DPP-4 Inhibitors in Japan

**DOI:** 10.3390/pharmaceutics4030479

**Published:** 2012-09-19

**Authors:** Tomomi Kimura, Kazuhito Shiosakai, Yasuaki Takeda, Shinji Takahashi, Masahiko Kobayashi, Motonobu Sakaguchi

**Affiliations:** 1 Epidemiology, Janssen Pharmaceutical K.K., 2-5-3, Nishi-Kanda, Chiyoda-ku, Tokyo 101-0065, Japan; 2 Pro-Bono Pharmacoepidemiologists Committee in Japan, Japan; 3 Clinical Data and Biostatistics, Daiichi Sankyo Co. Ltd., 1-2-58, Hiromachi, Shinagawa-ku, Tokyo 140-8710, Japan; 4 Sales planning group, Japan Medical Research Institute Co. Ltd. 9-1 Marunouchi 1-chome, Chiyoda-ku, Tokyo 100-6739, Japan; 5 Nihon Chouzai Co. Ltd. 9-1 Marunouchi 1-chome, Chiyoda-ku, Tokyo 100-6737, Japan

**Keywords:** Sulfonylurea, DPP-4 inhibitors, hypoglycemia, evaluation of risk minimization action, pharmacy claims, labeling change

## Abstract

After the launch of dipeptidyl peptidase-4 (DPP-4), a new oral hypoglycemic drug (OHD), in December 2009, severe hypoglycemia cases were reported in Japan. Although the definite cause was unknown, co-administration with sulfonylureas (SU) was suspected as one of the potential risk factors. The Japan Association for Diabetes Education and Care (JADEC) released a recommendation in April 2010 to lower the dose of three major SUs (glimepiride, glibenclamide, and gliclazide) when adding a DPP-4 inhibitor. To evaluate the effectiveness of this risk minimization action along with labeling changes, dispensing records for 114,263 patients prescribed OHDs between December 2008 and December 2010 were identified in the Nihon-Chouzai pharmacy claims database. The adherence to the recommended dosing of SU co-prescribed with DPP-4 inhibitors increased from 46.3% before to 63.8% after the JADEC recommendation (*p* < 0.01 by time-series analysis), while no change was found in those for SU monotherapy and SU with other OHD co-prescriptions. The adherence was significantly worse for those receiving a glibenclamide prescription. The JADEC recommendation, along with labeling changes, appeared to have a favorable effect on the risk minimization action in Japan. In these instances, a pharmacy claims database can be a useful tool to evaluate risk minimization actions.

## 1. Introduction

Dipeptidyl peptidase-4 (DPP-4) inhibitors are the most recently approved class of agent for Type 2 diabetes therapy [1]. Their efficacy and safety, both in monotherapy and combination therapies with other antidiabetics, have been reviewed in many clinical trials [2,3].

In Japan, four DPP-4 inhibitors were available as of December 2011: sitagliptin, vildagliptin, alogliptin, and linagliptin (since December 2009, April 2010, June 2010, and September 2011, respectively) as of March 2012. They can be used as monotherapy or in combination with sulfonylureas (SU), biguanides, alpha-glucosidase inhibitors, or glitazones.

Hypoglycemia is one of the important safety concerns with oral antidiabetic therapies [4]. Placebo-controlled trials with Japanese diabetes patients have shown that DPP-4 inhibitors are not likely to cause hypoglycemia [5,6,7,8,9,10,11,12,13,14,15,16]. However, with SU combination therapy, there was a small concern of an increased risk of hypoglycemia, although no severe hypoglycemia was reported in the clinical trials [12,13,14,15,17]. A 12-week clinical trial of combination therapy with sitagliptin and glimepride showed a tendency to increase the frequency of hypoglycemia (5.6% (4/71) in the sitagliptin group and 0% (0/67) in the placebo group), compared to that in monotherapy or combination therapy with other oral hypoglycemic drugs (OHDs) (0%–3.0%) [12]. In another 12-week clinical trial that compared vildagliptin 50 mg twice-daily with placebo when added to glimepride (≥1.0 mg/day), the incidence of hypoglycemic events was 2.0% (2/102) in the vildagliptin group compared to 1.0% (1/100) in the placebo group [13,17]. Similar results were shown in a 12-week clinical trial of combination therapy between alogliptin and glimepride (1.9% (2/104) in the alogliptin group and 0.97% (1/103) in the placebo group) [15]. Since the launch of the first DPP-4 inhibitor, severe hypoglycemia has been reported in Japan, including 32 cases of serious hypoglycemia in patients receiving sitagliptin reported within six months after the approval [18], the majority of which (28/32) used an SU concomitantly in spite of cautions of hypoglycemia in concomitant use with SU in the sitagliptin labeling. Although the definite cause was unknown, the Japan Association for Diabetes Education and Care (JADEC) released a “Recommendation” on 7 April 2010 to promote proper co-administration of a DPP-4 inhibitor and an SU [19]. In the same month, similar instructions were added in the package insert for all DPP-4 inhibitors as a part of class labeling. Consequently, the reporting rate of serious hypoglycemia for sitagliptin declined from 25 reports/month in April 2010 to 5 in June 2010 [20]. However, because it is not clear if the reporting rate directly reflects the reduction of SU doses according to the recommendation, further analysis was required to evaluate the effectiveness of these risk minimization actions.

Preliminary analyses in our and other studies, using an independent pharmacy database, showed similar results: the proportion of SU prescriptions appropriately reducing their dose when added to a DPP-4 inhibitor increased after the recommendation release [21,22]. In the current study, we looked at SU combination therapies with other OHDs than DPP-4 inhibitors to evaluate the effectiveness of the JADEC recommendations and labeling changes with regard to the specific impact on the SU + DPP-4 inhibitor co-prescriptions. Furthermore, we identified any differences among the SU products themselves. 

## 2. Methods

### 2.1. Data Source and Dispensing Practice in Japan—Pharmacy Claims

In Japan, outpatients need to see a physician every time they need a prescription, which is valid only for four days. Prescriptions are collected at the pharmacy upon dispensing and cannot be refilled; therefore, it is generally believed that the prescription and dispensing records are essentially equivalent in Japan. The drug price is standardized nationwide and patients can choose any pharmacy regardless of their insurance programs. There are two ways of dispensing: intramural (dispensed at the medical institution where the prescription was issued) and extramural (dispensed at one of the community pharmacies of the patient’s choice). Extramural dispensing accounted for 70.1% of the total prescriptions for outpatients from hospitals and 60.2% of those from general practitioners in May 2010 [23]. Nihon-Chouzai is the second largest nationwide pharmacy chain in Japan and provides a large database containing extramural dispensing records for 7,100,000 unique patients from 40,809 medical institutes since April 2001. Currently, 362 Nihon-Chouzai pharmacies handle approximately 1.2% of extramural prescriptions in Japan for 1,930,000 outpatients annually with a mean follow-up of 3.8 years [24]. Although it captures only those outpatients with extramural dispensing, the age distribution of the patients in our dataset (any users of antidiabetics) was similar to that among nationwide outpatients diagnosed with diabetes based on a self-reported health questionnaire survey of approximately 290,000 households and 750,000 household members who were randomly selected by a stratified random sampling method [25]. No clinical data were available in the dispensing claims database and it was not possible to capture the hypoglycemia episodes.

Pharmacy dispensing records for antidiabetics between 1 December 2008 and 31 December 2010 were extracted from the Nihon-Chouzai pharmacy claims database by one of the study authors (S.T.) and included the scrambled patient identification number (ID), age on the 1st day of the month of dispensing, sex, prescription ID, drug identification code (YJ code), dispensing date, medication frequency of administration per day, number of beds in the prescriber’s institute (according to the Nihon-Chouzai internal record as of December 2010), and scrambled institute ID. The YJ codes for each antidiabetic were as follows: SU (the first 4 digits are 3961 or 3961XXX), DPP-4 inhibitors (3969010, 3969011, and 3969012), insulin (2942XXX), alpha-glucosidase inhibitors (3969003, 3969004, and 3969009), biguanides (3962XXX), glitazones (3969005 and 3969007), glucagon-like peptide-1 (GLP-1) analogues (2499410), glinides (3969006 and 3969008), and a fixed-dose combination of glitazone and biguanide (3969100). 

### 2.2. SU Daily Dose

The JADEC recommendation proposed that the SU dose should be lowered to the following target doses when adding a DPP-4 inhibitor: ≤2.0 mg/day for glimepride, ≤1.25 mg/day for glibenclamide, and ≤40 mg/day for gliclazide. We calculated the SU daily dose by multiplying the dose per tablet, which can be obtained from the YJ code, and the number of tablets prescribed per day. The records for 94 patients with two or more different SU products (e.g., glimepiride and glibenclamide) on the same day were excluded, since no instruction was listed in the recommendation. The daily SU dose for another 841 pairs of prescriptions with multiple records of the same SU product on the same day for the same prescription days was determined as the sum of their doses. For example, a pair of same-day prescriptions of 1.0 mg/day of glimepiride and 3.0 mg/day of glimepiride for 28 days was interpreted as one prescription of 4.0 mg/day of glimepiride for 28 days. When there were multiple records of SU prescription with different doses and number of prescription days, we interpreted them individually by carefully examining each record and the Nihon-Chouzai internal log. Nihon-Chouzai pharmacists confirmed the physician’s intention by phone when they had any questions before dispensing and noted the specific instruction from the physician in an internal log. In this study, these logs were confirmed by one of the study investigators (Y.K.). 

### 2.3. Prescription Duration

Prescription duration was obtained from the dispensing record. For 31 pairs of prescriptions with multiple records of the same SU product on the same day and different prescription days, we added the prescription days. For example, two prescriptions for 1.0 mg/day of glimepiride for 28 days and for 7 days, written on the same day, were interpreted as one glimepiride prescription for 1.0 mg/day for 35 days. When there were multiple records of SU prescriptions with different doses and prescription days, we interpreted the data individually. On assessing the prescription days for DPP-4 inhibitors, we found a “pulse” pattern, characterized by (1) a prescription for 14 days followed by another 14 days of prescription with a 12–16 day interval between the two prescriptions; (2) administration twice or more times per day; and (3) this pattern repeated consecutively two times or more. In Japan, prescription days are limited to two weeks right after the launch of any new product until the 1st day of the 13th month from the launch. DPP-4 inhibitors were under this restriction during the study period and therefore, the prescription days for records that met the above-mentioned criteria were interpreted as 28 days of continuous therapy, rather than a repeated on-and-off prescription every two weeks. 

### 2.4. Evaluation of Risk Minimization Activities

The number of prescriptions was calculated as the number of the dates on which a prescription was dispensed per patient. Co-prescription was defined as a same-day prescription. For subgroup analyses, SU co-prescriptions with a DPP-4 inhibitor were counted as SU + DPP-4 inhibitor, regardless of other OHD co-prescriptions. Same-day co-prescriptions of SU with alpha-glucosidase, biguanide, glitazone, and other OHDs (including glucagon-like peptide-1 analogues (GLP-1), glinide, and combination drugs of glitazone and biguanide), but without DPP-4 inhibitors, were counted for each: For example, a same-day co-prescription of SU + alpha-glucosidase + biguanide was included both in the SU + alpha-glucosidase and in the SU + biguanide subgroups. 

In order to evaluate the impact of the JADEC recommendation released on 7 April 2010, two outcome markers were applied: the proportion of SU + DPP-4 inhibitor same-day co-prescriptions (for all subclasses) and the mean daily dose of SU (for each subclass). The change in adherence (the proportion of the SU prescriptions under the recommended dose) with or without same-day DPP-4 inhibitor prescriptions was assessed by segmented regression analysis of interrupted time series (time series analysis) [26]. Time series analysis is a method, which can be applied to see whether an intervention affects subsequent observations. The regression model treated the proportion of the SU prescription that were compliant with the JADEC recommendation in each 10-day interval as the outcome variables, and the following three as the predictor variables: (1) the time after the first DPP-4 inhibitor launch; (2) an indicator variable for occurring before (indicator = 0) or after (indicator = 1) the JADEC recommendation release, and (3) the time after the recommendation release. Given one month of lag time where labeling changes and manufacturers’ activities took place after the JADEC recommendation release, we tested another model by excluding the outcome variables during the lag time. 

The mean daily dose of each SU subclass (glimepiride, glibenclamide, and gliclazide) was calculated for SU monotherapy (SU without having any same-day OHD co-prescriptions), SU with a same-day OHD co-prescription but not with DPP-4 inhibitors, and for SU + DPP-4 inhibitor same-day co-prescriptions. The changes between the periods before (11 December 2009 to 10 April 2010) and after (11 April to 31 December 2010) the recommendation release were compared using the segmented regression analysis model, treating the mean SU dose by each SU subclass in each 10-day interval as the outcome variable. In addition, the chronological changes in the mean daily doses by 10-day interval were also descriptively compared among the SU monotherapy, SU + DPP-4 inhibitor, and SU + other OHDs. 

The impact of the recommendation was also analyzed according to the patients’ age (under 65 year-old *vs.* 65 year-old or older) and by the size of the medical institutions where the drugs were prescribed. The size of the institutions was categorized by the number of beds: <20, 20–399, and 400 or more. The number of beds in each medical institution was determined according to the Nihon-Chouzai internal record as of December 2010. The statistical significance level was defined as 5% and all tests were two-sided. All the analyses were performed using SAS^®^Release 9.2 (SAS Institute Inc., Carey, NC, USA).

## 3. Results

Our dataset included dispensing records for 114,263 patients, of which 48.5% (*n* = 55,426) were prescribed an SU at least once during the study period (1 December 2008 and 31 December 2010). After the launch of the first DPP-4 inhibitor (11 December 2009), a total of 44,866 patients were prescribed SUs with or without a DPP-4 inhibitor (Table 1). Almost all the patients (99.1%) received all of their antidiabetic prescriptions from a single medical institution. The patients co-prescribed an SU with DPP-4 inhibitor at least once during this period were younger compared to those who never had a same-day DPP-4 inhibitor and had more frequent dispensing (*p* < 0.01). The length of prescription days per dispensing for SUs was much longer than that for DPP-4 inhibitors (mean: 40.7 *vs.* 16.4 days, median 35.0 *vs.* 14.0 days). 

**Table 1:** Characteristics of sulfonylureas (SU) users with or without same-day dipeptidyl peptidase-4 (DPP-4) inhibitor.

	SU with same-day DPP-4 inhibitor	SU without same-day DPP-4 inhibitor
Patient, *N*	2,762	42,104
Male, %	59.9	61.5
Age *, mean (SD)	63.9 (12.2)	66.2 (12.0)
65 year or older, %	49.7	57.8
*N* of dispense per patient, mean (SD) **	9.4 (6.6)	6.1 (4.1)
*N* of prescription sites per patient, median (range)	1 (1–2)	1 (1–3)

Data between December 11, 2009 and December 31, 2010 (about 13 months); DPP-4, Dipeptidyl peptidase-4; SD, standard deviation; * Age at the first prescription for the patient during this period; ** Counted as the number of dispense dates.

**Figure 1:**
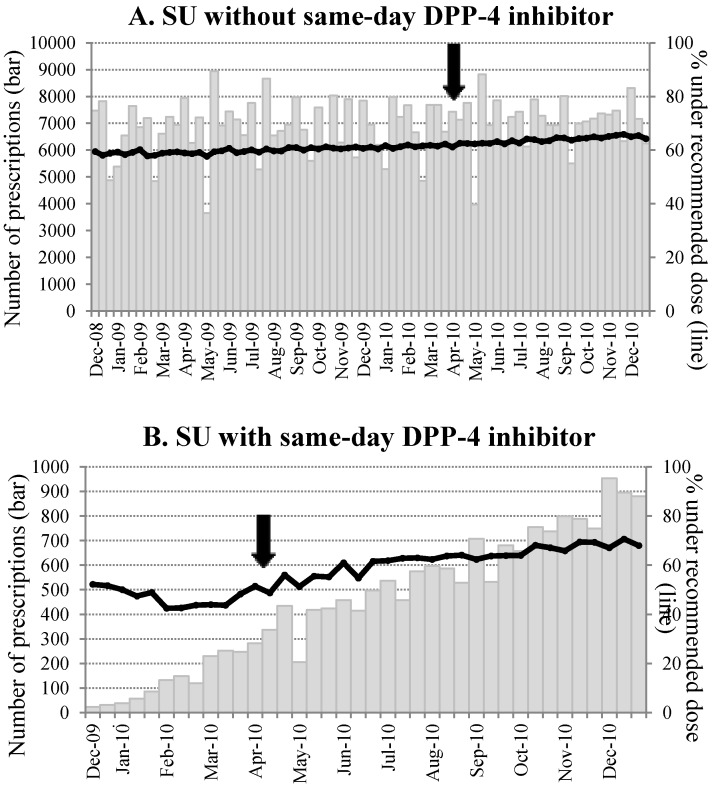
Chronological trend in number of SU prescription (bars)and proportion to SU prescriptions under the recommended dose (lines). (**A**) SU prescriptions without DPP-4 inhibitor; (**B**) SU prescriptions with DPP-4 inhibitor.Data is summarized for every 10 days (1st to 10th, 11th to 20th, and 21t to the end of the month for each month). The arrows indicate the timing of the recommendation release.

In the SU prescriptions without a same-day DPP-4 inhibitor, there was no significant change in the number of prescriptions throughout the study period (average ~7,000 for every 10 day period). On the other hand, the number of SU prescriptions with a same-day DPP-4 inhibitor increased rapidly from the time of the launch (Figure 1). Before the recommendation, 46.3% of SU prescriptions co-prescribed with a DPP-4 inhibitor were compliant with the recommendation. The proportion increased from 17.5% to 63.8% after the recommendation (*p* for a difference < 0.01). The time-series analyses demonstrated that the recommendation was significantly and favorably associated with subsequent SU prescription practice: The proportion of SU prescriptions under the recommended SU dose was around 50% after the first DPP-4 inhibitor launch (December 2009) and it increased to 68% after eight months from the recommendation (at the end of 2010, *p* = 0.0003). The slopes of the proportion of adherent prescription over time in the periods before and after the recommendation were significantly different (estimate (standard error) −0.39 (0.24) *vs.* 0.68 (0.08), *p* = 0.0002). On the other hand, the recommendation was not significantly associated with the SU doses in mono-prescriptions (*p* = 0.36). Similar results were found among elderly (65 year old or older) and younger patients (data not shown). Allowing for one month of lag time during which labeling changes and manufacturers’ activities took place after the JADEC recommendation release, the results remained essentially the same as in the original model. In addition, our sub-analysis demonstrated that there was no difference in the chronological trends and in the increased adherence before/after the recommendation release when stratifying by the size of the medical institution (data not shown).

The proportion of SU prescriptions compliant with the recommendation varied across the type of SU. Glimepiride was the major SU with (81%) or without (71%) a DPP-4 inhibitor and the adherence to recommended dosing at baseline (53.2%) increased to 69.4% after the recommendation. Gliclazide accounted only for 7% and 10% with and without a DPP-4 inhibitor, respectively, and its compliance with the recommendation was the highest among the three SUs (66.4% in pre-recommendation and 76.7% in post-recommendation). The third SU, glibenclamide, showed substantially different results: only 4.9% (13/266) and 16.4% (295/1796) were compliant in pre-recommendation and post-recommendation periods when co-prescribed with a DPP-4 inhibitor. 

In total, 65.4% of the 536,500 SU prescriptions were co-prescribed with OHDs. In the SUs without a same-day DPP-4 inhibitor co-prescription, alpha-glucosidase inhibitors were most frequently co-prescribed (38.6%), followed by biguanide (30.1%), and glitazone (25.6%). SU co-prescription patterns before and after the first DPP-4 inhibitor launch are shown in Table 2. After the launch of the first DPP-4 inhibitor in Japan, we identified 17,250 (6.1%) and 265,987 SU prescriptions with and without a same-day DPP-4 inhibitor. The proportion of SU monotherapy decreased to 4% after the launch whereas that for co-prescriptions with the other OHDs was almost stable. Among the 17,250 same-day SU + DPP-4 inhibitor co-prescriptions, 63.7% were also co-prescribed with another OHD, including alpha-glucosidase inhibitors (16.7%), biguanide (42.6%), and glitazone (29.4%). 

**Table 2:** SU and same-day co-prescription before and after the DPP-4 inhibitor launch in Japan.

	Before launch ^a^	After launch ^b^
SU Prescription, *N*	253,263	283,237
SU monotherapy	33.8	29.8
With Insulin	5.0	5.7
With OHDs	64.5	66.2
DPP-4 inhibitor	--	6.1
Alpha-glucosidase inhibitor *	38.5	36.3
Biguanide *	28.7	29.5
Glitazone *	25.1	24.5
Others ^c^^,^*	0.6	0.7

% where not otherwise indicated. Number of SU prescriptions was calculated as the sum of dates where SU was prescribed per patient. DPP-4, Dipeptidyl Peptidase-4; OHD, oral hypoglycemic drug; * without having same-day DPP-4 inhibitors; ^a^ 1 December 2008 to 10 Decmber 2009; ^b^ 11 December 2009 to 31 December 2010; ^c^ Others include glucagon-like peptide-1 analogues (GLP-1), glinide, and combination drug of glitazone and biguanide.

Table 3 shows the changes in the SU mean doses before and after the recommendation release either in SU monotherapy or by same-day OHD co-prescription. Glimepiride and glibenclamide were used more with alpha-glucosidase inhibitors than as monotherapies, while gliclazide was used mainly as its monotherapy. The average SU doses were 30%–40% lower when used as monotherapy in all the SU subclasses. Glibenclamide tended to be used in higher doses than the recommended dose for DPP-4 inhibitor co-prescription (1.25 mg/day), even for the monotherapy. In the four-month period between the launch of the first DPP-4 inhibitor and the recommendation release, the mean daily dose was highest in glimepiride and glibenclamide with a DPP-4 inhibitor co-prescription, while it was lower in gliclazide compared to the co-prescriptions with the other OHDs. After the recommendation release, the average daily dose of glimepiride and glibenclamide co-prescribed with a DPP-4 inhibitor declined by 20.6% and 24.0%, respectively, while that for gliclazide was similar (1.9% decrease). Slight decreasing trends were found in SU monotherapies (0.3%–2.7%) and same-day prescriptions of SU with the other OHDs (1.0%–4.1%), but the difference did not reach a statistically significant level in the time-series analysis.

**Figure 2:**
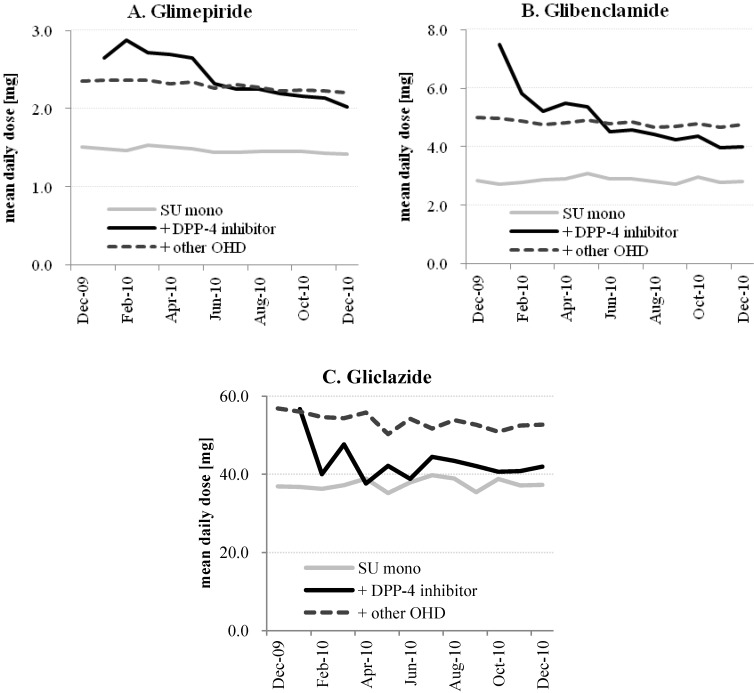
Chronological trend in SU mean daily dose with or without same-day DPP-4 inhibitor and the other oral hypoglycemic drugs (OHD). The mean daily dose of glimepiride (**A**) glibenclamide; (**B**) gliclazide; (**C**) was calculated for every 10 days. Gray lines indicate the SU monotherapy, black lines indicate the SU with same-day DPP-4 inhibitor, and dotted lines indicate SU with same-day OHDs but not with DPP-4 inhibitor.

The chronological changes in the mean daily SU dose are illustrated in Figure 2. Without a same-day DPP-4 inhibitor (either SU monotherapy or SU + other OHDs), the daily dose was consistent throughout the study period. When prescribed with a DPP-4 inhibitor, the mean daily dose decreased after the JADEC recommendation for all SU subclasses. The mean daily dose for glimepiride + DPP-4 inhibitor was higher than those without a same-day DPP-4 inhibitor before the recommendation release but decreased gradually after the recommendation release, approaching the recommended dose (2.0 mg/day) at the end of the study period. The glibenclamide average daily dose was also higher when a DPP-4 inhibitor was co-prescribed and decreased after the recommendation, but was still 3.3 times higher than the recommended dose (1.25 mg/day), even eight months after the recommendation release. Gliclazide use with a DPP-4 inhibitor was fairly limited but the daily dose became lower than that for gliclazide + other OHDs. 

**Table 3:** Change in SU dose before and after the DPP-4 inhibitor launch and before and after the Japan Association for Diabetes Education and Care (JADEC) recommendation release by type of co-prescribed antidiabetics.

	Between launch and recommendation ^a^		Post-Recommendation^ b^		% change in pre-/post Recommendation
	*N* of prescription	Mean dose (mg)	*N* of prescription	Mean dose (mg)	
Glimepiride monotherapy	18,227	1.49	41,090	1.45	−2.7
+ Alpha-GI *	23,138	2.45	52,412	2.35	−3.8
+ Biguanide *	18,932	2.48	45,132	2.38	−4.1
+ Glitazone *	15,989	2.59	36,319	2.50	−3.5
+ DPP-4 inhibitor	1,260	2.76	12,715	2.19	−20.6§
Glibenclamide monotherapy	4,306	2.85	8,651	2.83	−0.9
+ Alpha-GI *	6,674	4.92	13,502	4.78	−2.9
+ Biguanide *	4,221	5.19	8,616	5.13	−1.2
+ Glitazone *	4,229	5.18	8,361	5.07	−2.1
+ DPP-4 inhibitor	266	5.70	1,796	4.33	−24.0
Gliclazide monotherapy	3,955	37.8	8,205	37.7	−0.3
+ Alpha-GI *	2,255	55.4	4,880	54.5	−1.6
+ Biguanide *	2,043	55.9	4,635	54.2	−2.9
+ Glitazone *	1,431	55.2	3,176	54.7	−1.0
+ DPP-4 inhibitor	119	45.3	1,094	44.4	−1.9

Number of SU prescriptions with the other antidiabetics is indicated as the number of same-day co-prescriptions. Alpha-GI, Alpha-glucosidase inhibitor; DPP-4, Dipeptidyl Peptidase-4; * without having same-day DPP-4 inhibitors; ^a^ Dec 11, 2009 to Apr 10, 2010; ^b^ 11 Apr 2010 to 31 Dec 2010; § *p* < 0.01 by the time-series analysis.

## 4. Discussion

In this study, we evaluated the effectiveness of risk minimization activities in Japan intended to avoid severe hypoglycemia by reducing the SU daily dose when adding a DPP-4 inhibitor. Using secondary dispensing data, we demonstrated that the SU dose has been successfully reduced for combination use with a DPP-4 inhibitor. Although the adherence for combinations with other OHDs was also slightly improved (not significantly) after the recommendation, this might be affected by increased caution about the risk of hypoglycemia for all SU users due to the recommendation, which was specifically aimed at users of an SU co-administered with a DPP-4 inhibitor. The specific change for combination with a DPP-4 inhibitor suggests that the change was attributable to the recommendation release and consequent labeling changes, rather than other factors. The adherence at the end of the study period (after eight months from the recommendation) did not reach 100%. However, the treatment for individual patients is left to the discretion of the attending physician, who might prescribe a higher dose SU with a DPP-4 inhibitor for improvement in glycemic control while carefully watching the risk of hypoglycemia.

In Japan, several pharmacovigilance activities are available: Early Postmarketing Phase Vigilance, which is a system to enhance adverse event reporting within six months after the launch of a new product [27], and post-marketing surveillance to evaluate the safety and effectiveness in the general population. The severe hypoglycemia cases were reported from such post-marketing programs. However, these programs usually target the patients who are exposed to a particular product and therefore have no data on comparison groups. Also, risk minimization actions (e.g., dear doctor letters, labeling changes) have been performed for each safety concern; in this case, cautions of hypoglycemia in concomitant use with SU were indeed included in the labeling of sitagliptin. However, the dose reduction of the SU was not specifically instructed. The quantitative evaluations of effectiveness of such actions remain scarce in Japan [28,29,30], while claims data have been used skillfully for the evaluations in Western countries [31,32,33,34,35,36,37,38]. Because the Risk Management Plan (RMP) containing requirements for the evaluation of risk minimization actions has been implemented in Japan since April 2012, our study could be a meaningful precedent for similar interventions and the quantitative evaluation using a pharmacy claims database in Japan.

SUs are the most frequently used OHDs in Japan [39] and DPP-4 inhibitors are most likely to be co-prescribed with SUs (47.8%). Our data showed that the mean SU dose in combination therapy with a DPP-4 inhibitor before the recommendation release was higher than in SU monotherapy or in combination with other OHDs (Figure 2). The reason might be that a DPP-4 inhibitor was added in the patients with secondary failure who were already using a high-dose of SU. It is worth noting, however, that a reduction in the dose of SU according to the recommendation and labeling was usually performed when a DPP-4 inhibitor was added, to reduce the risk of severe hypoglycemia. 

Each SU investigated showed a reduction of the mean daily dose after the recommendation release. However, the mean daily dose for glibenclamide did not reach the recommended reduction in dose (1.25 mg/day). The recommended dose for glibenclamide might be conservative because glibenclamide has been reported to have a higher risk of hypoglycemia than other SUs [40,41].

The adherence to the recommended dosing appears to have increased gradually since February 2010, even before the recommendation release in April 2010. One of the reasons might be an impact of the change in US labeling of sitagliptin in February 2010 [42] and the likely increased discussion about hypoglycemia risk in the medical community globally. The revision added cautions about an increased risk of hypoglycemia when combining the use of sitagliptin with an SU or insulin and suggested lowering the dose of the SU or insulin. The other possible reason is an influence of the size of the medical institution (e.g., clinics with <20 beds or large hospitals with ≥400 beds). The proportions of institutions by size were consistent over time for SU prescriptions without same-day DPP-4 inhibitors: ten to fifteen percent of clinics and 35%–40% of large hospitals. On the other hand, in the group of SU prescriptions with a same-day DPP-4 inhibitor, the major prescription sites were small clinics (>30%) for the first two months, whereas the proportion of large hospitals has gradually increased from 18% to 35% since the beginning of 2010. In addition, glibenclamide was likely to be prescribed with a DPP-4 inhibitor in clinics rather than large hospitals in our data. Therefore, the changes in the proportion of institution size might have led to the slight increase in the adherence rate from February 2010.

There are some limitations of this study. First, it was not possible to investigate whether actual hypoglycemia episodes were reduced after the recommendation release because no clinical data were available in the dispensing claims database. Second, since only concurrent prescriptions for an SU and a DPP-4 inhibitor were addressed as co-administration, there might be more patients co-administered by separate prescriptions. Third, because we captured the prescriptions only when the patient showed up at one of the Nihon-Chouzai pharmacies, no information was available for the patients who were dispensed drugs at different pharmacies. Also, even within this pharmacy chain, the intention of physician was not always clear: Our intention on interpreting the data was to minimize the gap between the end of the prescription days and the next prescription for each patient. The calculation of prescription days, a seven-day together with a 28-day prescription issued on the same day was counted as a 35-day prescription, which fit our purpose but it is also true that it could underestimate the daily SU dose if physicians actually indicated a higher dose for several days during the total prescription days. However, only 31 pairs of prescriptions fell into this category and we consider that the impact on the final result should be minimal. Indeed, a sensitivity analysis excluding these 31 pairs of prescriptions showed essentially the same results (data not shown). Finally, the pharmacy claims database does not include the prescriptions for inpatients. Also, about 30% of the prescriptions from hospitals and 40% from general practitioners were dispensed at the medical institution and could not be observed [23]. Some medical institutions promote extramural dispensing while the others recommend intramural dispensing leaving the final decision to patients. Therefore, our dataset may or may not represent the prescriptions from the latter type of medical institutions. Nonetheless, we consider this study still meaningful because it would be more challenging to monitor hypoglycemia events in outpatients, compared to inpatients, and the recommendation was for the general practitioners, rather than for diabetologists. In addition, another study conducted independently with another pharmacy claims database showed a very similar adherence level, although they investigated only the combination therapy with glimepride and sitagliptin [22].

## 5. Conclusions

The results of this study strongly suggest that the JADEC recommendation, along with labeling changes, was effective in reducing daily SU doses in combination therapy with a DPP-4 inhibitor in clinical practice. We assessed SU combination therapies with other OHDs and showed that there was an exclusive impact of the recommendation on the co-prescriptions of SU and DPP-4 inhibitors. Correlation between the change in prescription pattern and the decrease in number of hypoglycemia episodes needs to be assessed in subsequent research to determine whether the change in prescribing had the desired impact on the risk of hypoglycemia. 
